# Melatonin inhibiting the survival of human gastric cancer cells under ER stress involving autophagy and Ras‐Raf‐MAPK signalling

**DOI:** 10.1111/jcmm.16237

**Published:** 2020-12-25

**Authors:** Yongye Huang, Kexun Yuan, Meifang Tang, Jiaming Yue, Lijun Bao, Shuang Wu, Yanxin Zhang, Yin Li, Yihang Wang, Xu Ou, Jiaxin Gou, Qi Zhao, Lin Yuan

**Affiliations:** ^1^ College of Life and Health Sciences Northeastern University Shenyang China; ^2^ National Academy of Innovation Strategy Beijing China; ^3^ School of Computer Science and Software Engineering University of Science and Technology Liaoning Anshan China; ^4^ Institute of Health Science China Medical University Shenyang China

**Keywords:** autophagy, combinatorial treatment, ER stress, melatonin, RNA‐seq

## Abstract

Melatonin exhibits antitumour activities in the treatment of many human cancers. In the present study, we aimed to improve the therapeutic potential of melatonin in gastric cancer. Our results confirmed that melatonin dose‐dependently suppressed the proliferation and necrosis, and increased G0/G1 phase arrest, apoptosis, autophagy and endoplasmic reticulum (ER) stress. The Ras‐Raf‐MAPK signalling pathway was activated in cells after melatonin treatment. RNA‐seq was performed and GSEA analysis further confirmed that many down‐regulated genes in melatonin‐treated cells were associated with proliferation. However, GSEA analysis also indicated that many pathways related to metastasis were increased after melatonin treatment. Subsequently, combinatorial treatment was conducted to further investigate the therapeutic outcomes of melatonin. A combination of melatonin and thapsigargin increased the apoptotic rate and G0/G1 cell cycle arrest when compared to treatment with melatonin alone. Melatonin in combination with thapsigargin triggered the increased expression of Bip, LC3‐II, phospho‐Erk1/2 and phospho‐p38 MAPK. In addition, STF‐083010, an IRE1a inhibitor, further exacerbated the decrease in survival rate induced by combinatorial treatment with melatonin and thapsigargin. Collectively, melatonin was effective in gastric cancer treatment by modifying ER stress.

## 1 INTRODUCTION

Gastric cancer is the sixth most common malignancy and results in the third highest cancer‐related deaths worldwide.^1^ Despite advances in pathogenesis and clinical studies in recent years, the overall prognosis remains unsatisfactory. Cancer cell resistance to anticancer therapeutics is a major obstacle to cancer therapy. Therefore, the development of anticancer agents is primarily aimed to induce cell death of cancer cells. There are three known types of cell death, including apoptosis, autophagic cell death and necrosis.^2^ Apoptosis, an essential cell process in the homeostasis of a multicellular organism, is tightly controlled by ‘programed’ signalling, and normally occurs during development and aging. Cellular stress, including endoplasmic reticulum (ER) stress and DNA damage, actively induces apoptosis when cells are damaged beyond repair.^3^ During a period of stress, autophagy is another cellular process that enables cells to remove long‐lived or damaged cellular organelles and proteins by a controlled recycling pathway.^4^ Autophagy primarily acts as a protective mechanism and hyperactivation of autophagy can lead to cell death. Autophagic cell death is recognized by the appearance of large intracellular vesicles and in control of the autophagy machinery.^2^ Necrosis or necrotic cell death engages a wide variety of cell death processes and is characterized by the loss of plasma membrane integrity followed by cytoplasmic leakage.^5^ These types of cell death have been shown to be involved in cancer therapy. Therefore, modulating cell death would be beneficial to therapeutic outcome.

Adjuvant therapies that contain low‐cost natural products and show limited side effects have attracted significant interest.^6^ Melatonin, a hormone that is synthesized and secreted by the pineal gland mainly during the night, is a promising adjuvant molecule with many potential beneficial consequences in cancer treatment.^7^ In gastric cancer, melatonin has shown to inhibit epithelial‐to‐mesenchymal transition signalling mechanism by triggering ER stress.^8^ In fact, melatonin is a pleiotropic anticancer molecule that regulates tumour growth, angiogenesis, differentiation, metastasis and antitumour immunity to combat gastric cancer via multiple mechanisms.^9^ Studies that are aimed at the molecular mechanisms of melatonin may have great potential in cancer therapy.

Drug resistance and metastasis may develop during chemotherapy, thereby substantially compromising the therapeutic efficacy of cancer treatment. Combination cancer therapy would contribute to decrease acquired resistance, and improve the probability and magnitude of therapeutic responses. Melatonin has been shown to synergize the chemotherapeutic effect of 5‐fluorouracil via modifying many signalling pathways in colon cancer.^10^ Therefore, to further strengthen the application of melatonin in gastric cancer therapy, in this study, the synergized effect between anticancer drug and melatonin was also determined.

## 2 MATERIALS AND METHODS

### 2.1 Cell culture, drug preparation and cell transfection

Human gastric cancer cells SGC‐7901 were cultured in DMEM containing 10% foetal bovine serum, 1% glutamine, 1% non‐essential amino acids, 100 mg/mL streptomycin and 100 U/mL penicillin at 37°C in a 5% CO_2_ atmosphere. Melatonin was purchased from Sigma‐Aldrich, and the stock solution of melatonin was prepared in Dimethyl sulfoxide (DMSO).

### 2.2 Cell cycle analysis

After 24 hours of melatonin treatment, cells were collected using 0.1% trypsin in 2.5 mmol/L ethylenediaminetetraacetic acid and rinsed twice with ice‐cold PBS. Next, cells were fixed with 70% ethanol at −20°C overnight. Cells were then washed twice with ice‐cold PBS, and stained with PI/RNase Staining Buffer Solution (BD Biosciences) in the dark for 15 minutes. Then, cells were analysed by a flow cytometer (Fortessa, BD Biosciences). A histogram of the cell cycle distribution was generated from 20 000 events per sample and data were analysed using ModFit LT software.

### 2.3 Real‐time quantitative PCR (RT‐qPCR)

Total RNA was isolated using TRIzol reagent according to the manufacturer's guidelines. Reverse transcription was carried out using the All‐in‐One cDNA synthesis SuperMix (Bimake), and RT‐qPCR was performed using 2x SYBR Green qPCR Master Mix (Bimake) on a CFX96 Real‐time PCR detection system. For PCR amplification, glyceraldehyde phosphatedehydrogenase was used as an internal control to normalize gene expression levels.

### 2.4 Western blotting analysis

Gastric cells were washed three times in ice‐cold PBS, then harvested and lysed in cell lysis buffer containing a protease inhibitor cocktail. Lysates were separated on a 12% SDS‐PAGE gel and transferred to Polyvinylidene fluoride (PVDF) membranes. Membranes were blocked in 5% non‐fat milk solution, followed by incubation with primary antibody and 5% bovine serum albumin. After overnight incubation at 4°C, the appropriate HRP‐conjugated secondary antibody was added and incubated at room temperature for 2 hours. Protein bands were visualized using an ECL detection system.

### 2.5 Statistical analysis

Data are expressed as mean ± SEM, and statistical analyses were performed using SPSS software. Analyses of different treatment groups were conducted by one‐way ANOVA *P < 0.05* was considered statistically significant. Experiments were conducted in triplicate.

## 3 RESULTS

### 3.1 Melatonin‐inhibited gastric cancer proliferation by regulating cell cycle progression

**FIGURE 1:**
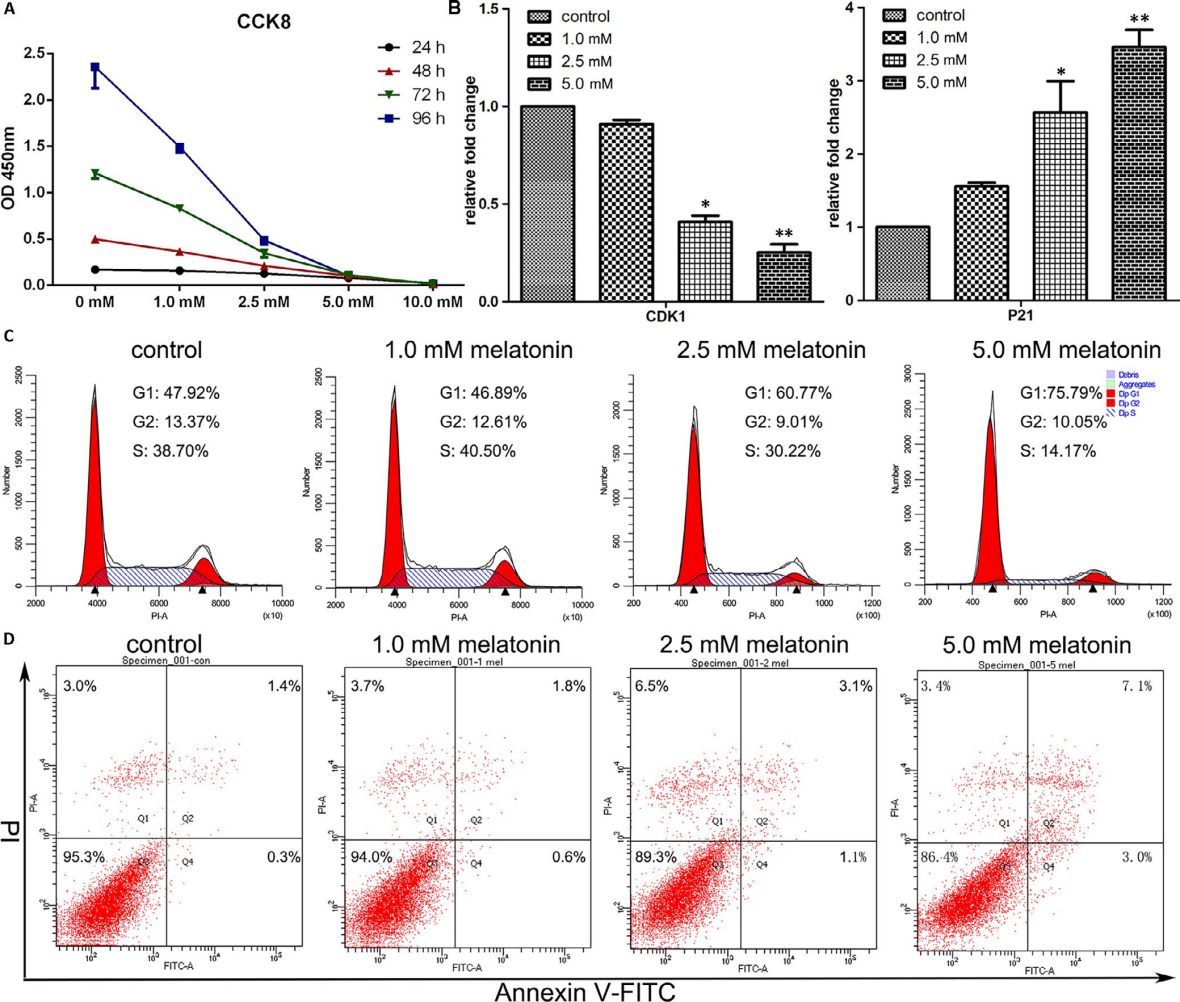
Melatonin affects the proliferation and cell cycle distribution of cancer cells. (A) The cell proliferation of cancer cells at different melatonin concentrations and time‐points of treatment. (B) Expression of CDK1 and P21 in cells after 24 h of melatonin treatment as determined by RT‐qPCR (mean ± SEM of duplicate experiments). *P < .05 versus control, and **P < .01 versus control. (C) Alterations in cell cycle distribution in untreated cells and cells treated with melatonin for 24 h were evaluated by flow cytometry using propidium iodide (PI) staining. (D) Apoptosis in the cells after 24 h of melatonin treatment was determined by flow cytometry analyses using double staining with Annexin V (horizontal line) and PI (vertical line)

Therapeutic compounds may affect cell viability according to the concentration and duration used, therefore, various concentrations of melatonin (0, 1.0, 2.5, 5.0 and 10.0 mmol/L) were used to determine gastric cancer cell viability at 24, 48, 72 and 96 hours. Treatment with melatonin‐inhibited cell proliferation in a dose‐ and time‐dependent manner (Figure 1A). To better investigate the underlying mechanism of melatonin treatment, the subsequent experiments were performed mainly using 0, 1.0, 2.5 and 5.0 mmol/L for 24 hours as exorbitant concentration and prolonged time duration inducing too serious cell death.

After treatment with 1.0, 2.5 and 5.0 mmol/L for 24 hours, the G0/G1 distribution dose‐dependently increased from 46.89%, 60.77% and 75.79%, respectively (Figure 1C), indicating treatment with a melatonin content triggered G0/G1 arrest in gastric cancer cells. To further determine the underlying mechanism involved in the induction of cell cycle arrest by melatonin treatment, the expression of CDK1 and p21 were evaluated by RT‐qPCR (Figure 1B). CDK1 expression, which is essential for G1‐S and G2‐M phase transitions, was significantly down‐regulated when melatonin concentrations increased to 2.5 and 5.0 mmol/L. Moreover, the expression of p21, a regulator of G1 cell cycle phase, was significantly enhanced by 2.5 and 5.0 mmol/L melatonin treatment.

### 3.2 Melatonin regulated many types of programmed cell death via ER stress

**FIGURE 2:**
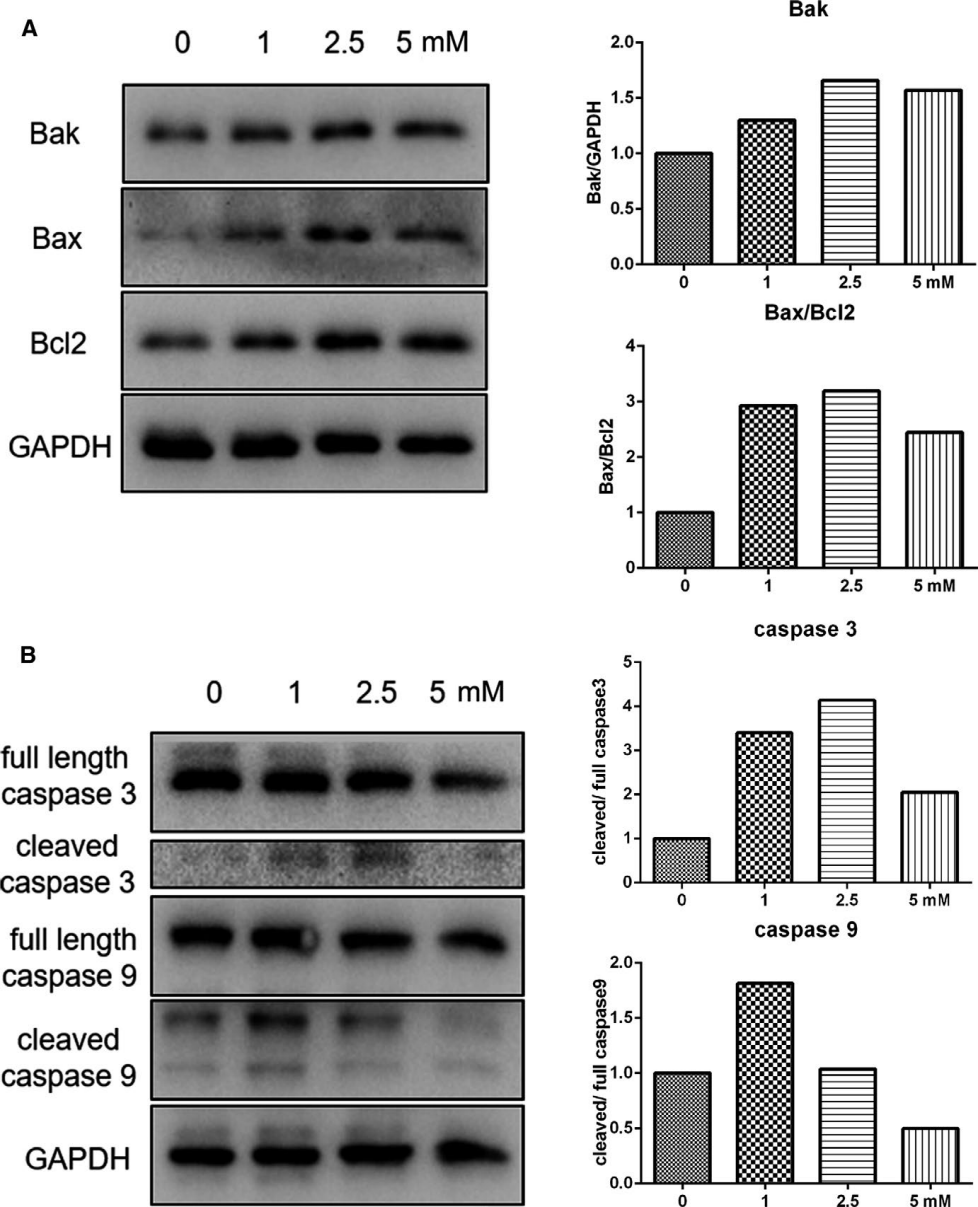
Evaluation of apoptosis in cells subjected to melatonin exposure for 24 h. (A) Expression of apoptosis‐associated genes as determined by Western blot analysis. (B) Expression of caspase 3 and 9 as determined by Western blot analysis

Different processes of cell death modulate each other by multiple feedback loops and influence the therapeutic outcome of chemotherapy. The canonical cell death modes include apoptosis, necrosis and autophagic cell death. In this study, the impact of melatonin treatment on apoptosis was first analysed. As revealed by flow cytometry using Annexin V‐FITC/PI staining, the apoptotic index in cells treated with 0, 1.0, 2.5 and 5.0 mmol/L melatonin was 1.7%, 2.4%, 4.2% and 10.1%, respectively (Figure 1D). Next, the expression of apoptosis‐associated genes, including FAS, HRK, TNFRSF10A and TNFRSF10B, was determined by RT‐qPCR (Figure S1). No significant differences were observed on the expression of FAS among the tested groups. The expression of HRK and TNFRSF10B was significantly increased by treatment with 1.0, 2.5 and 5.0 mmol/L melatonin, and the expression of TNFRSF10A was significantly increased in cells that were treated with 2.5 and 5.0 mmol/L melatonin. Subsequently, Western blot analysis was performed to confirm the expression of apoptosis‐associated genes. As shown in Figure 2A, Bak and Bax protein levels were up‐regulated by melatonin treatment. In addition, the ratio of Bax/Bcl2 was also enhanced in cells that were subjected to melatonin treatment. Apoptosis is a caspase‐dependent method of cell death. The increased expression of cleaved caspase 3 in melatonin‐treated cells was confirmed by Western blot analysis (Figure 2B). When compared with the control, the expression of cleaved caspase 9 was elevated in cells that were treated with 1.0 and 2.5 mmol/L melatonin, but not 5 mmol/L melatonin. The activation of caspase 3 was shown to be prevented by treatment with Z‐VAD‐FMK, a pan‐caspase inhibitor (Figure S2 and S3). However, Z‐VAD‐FMK did not enhance the survival rate of melatonin‐treated cells (Figure S4E).

Necroptosis is a type of programmed necrosis of which the signalling cascade is controlled by receptor‐interacting serine/threonine protein kinase 1 (RIPK1) or mixed‐lineage kinase domain‐like protein (MLKL). Results of RT‐qPCR analysis indicated that the expression of MLKL and RIPK1 was significantly increased by melatonin treatment (Figure S5A). Parthanatos is another type of controlled necrosis, which is triggered by hyperactivation of poly (ADP‐ribose) polymerase (PARP). However, no significant differences in the expression of PARP mRNA was observed in cells that were treated with melatonin when compared with the control (Figure S5A). Next, Western blot analysis was performed to evaluate the expression of PARP, and our findings confirmed that PARP showed a similar expression level among control cells and cells treated with different concentrations of melatonin (Figure S5B). Cleaved PARP increased in cells treated with melatonin in a dose‐dependent manner. Taken together, our data indicated that melatonin may possibly also subject gastric cancer cells into death via certain types of necrosis.

**FIGURE 3:**
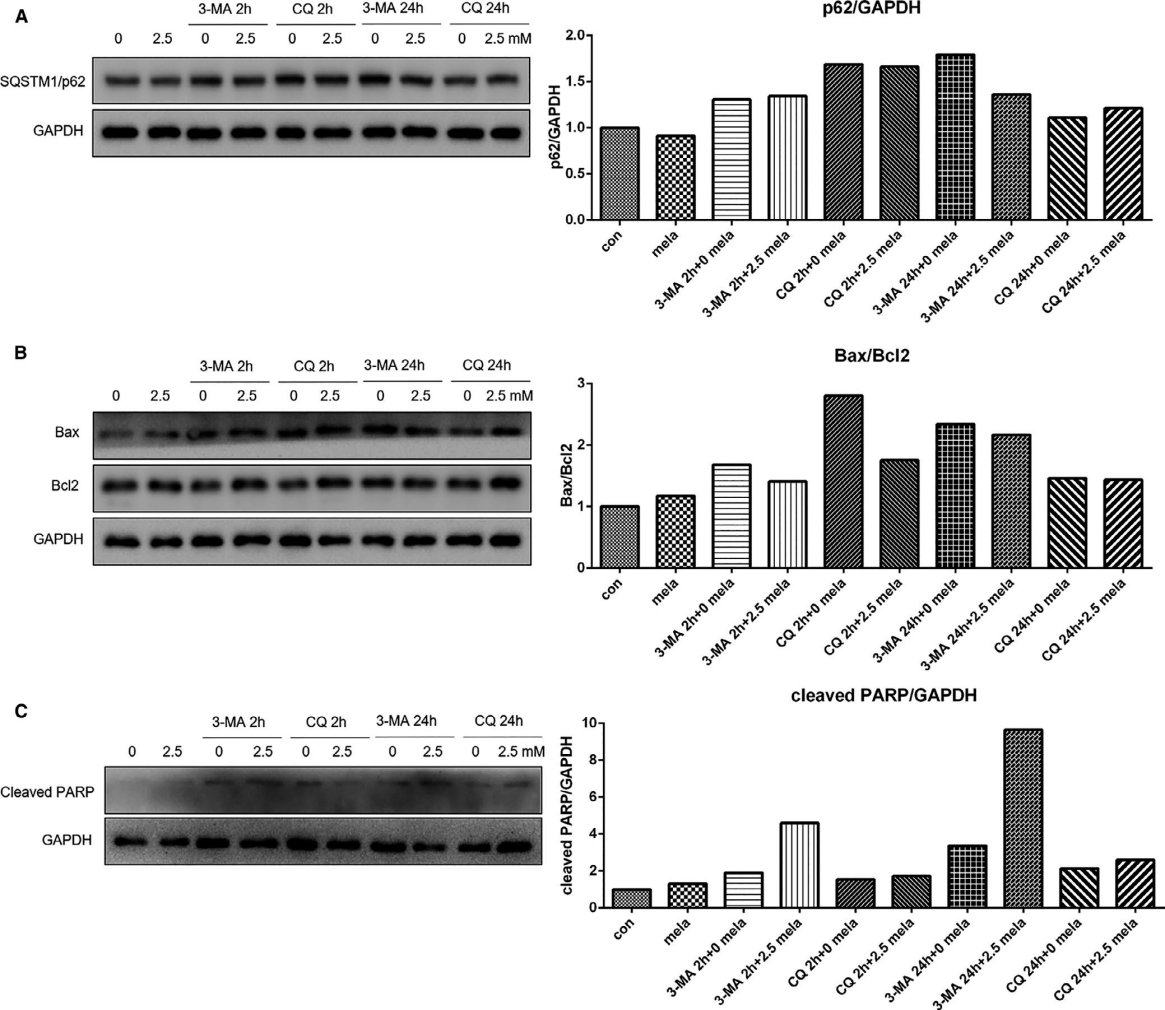
Expression of SQSTM1/p62 (A), Bax, Bcl2 (B) and cleaved PARP in cells pre‐treatment with 3‐MA (50 mmol/L) or CQ (20 μmol/L) for 2 h that were subsequently treated with melatonin (2.5 mmol/L) for 24 h, or cells that were simultaneously treated with melatonin (2.5 mmol/L) and 3‐MA (50 mmol/L) or CQ (20 μmol/L) for 24 h, as evaluated by Western blot analysis

Autophagy is well‐controlled process that plays a critical role in cellular homeostasis. As shown in Figure S6A, the mRNA expression of ATG3 and ATG5 was significantly up‐regulated in cells treated with 2.5 and 5.0 mmol/L melatonin. In addition, treatment with 5.0 mmol/L melatonin significantly increased the expression of Beclin1 at the transcriptional level, thereby indicating that autophagy was possibly induced by a high concentration of melatonin. To confirm this, Western blot analysis was conducted to determine the expression of LC3. Our results indicated that the accumulation of LC3‐II was significantly increased in a dose‐dependent manner (Figure S6B). Autophagy is controlled by complex signalling, and previous studies have shown that the PI3K/Akt/mTOR pathway plays important roles in the regulation of autophagy. As indicated in Figure S6C, treatment with melatonin reduced the phosphorylation level of Akt. Furthermore, the expression level of p‐mTOR was decreased in cells exposed to melatonin. Thus, these results indicated that autophagy was induced by melatonin and negatively associated with the PI3K/Akt/mTOR signalling pathway. In addition, Z‐VAD‐FMK could enhance the accumulation of LC3‐II in the cells treated with 2.5 mmol/L melatonin (Figure S2) but decreased LC3‐II expression in 5.0 mmol/L melatonin‐treated cells (Figure S3), indicating that there was crosstalk among different cell death mechanism under melatonin treatment. To further explore the role of autophagy in melatonin therapy, two autophagy inhibitors with different mechanisms were applied: 3‐Methyladenine (3‐MA) prevents the formation of autophagosome, and Chloroquine (CQ) neutralizes acidification of intracellular vesicles and inhibits degradation of the cargo in the autophagosomes.^11^ As shown in Figure 3A, the expression of SQSTM1/p62 was decreased by melatonin treatment but enhanced by 3‐MA or CQ treatment, confirming that melatonin‐induced autophagy could be blocked by 3‐MA or CQ treatment. The Bax/Bcl2 ratio was enhanced in the cells treated with melatonin, 3‐MA or CQ alone when compared with the control cells (Figure 3B). The increase in Bax/Bcl2 ratio in melatonin‐treated cells was decreased by 3‐MA or CQ therapy. However, CQ did not alter the survival rate of melatonin‐treated cells and 3‐MA even exacerbated the survival of melatonin‐treated cells (Figure S4C,D). We hypothesized that additional cell death mechanisms besides apoptosis were evoked in the cells treated with autophagy inhibitors in combination with melatonin. Therefore, the expression of cleaved PARP was determined via Western blot analysis. And cleaved PARP in melatonin‐treated cells was found to be further increased by 3‐MA or CQ therapy (Figure 3C). Combined, these results suggested that autophagy played an important role in the switch for different types of cell death during melatonin exposure.

**FIGURE 4:**
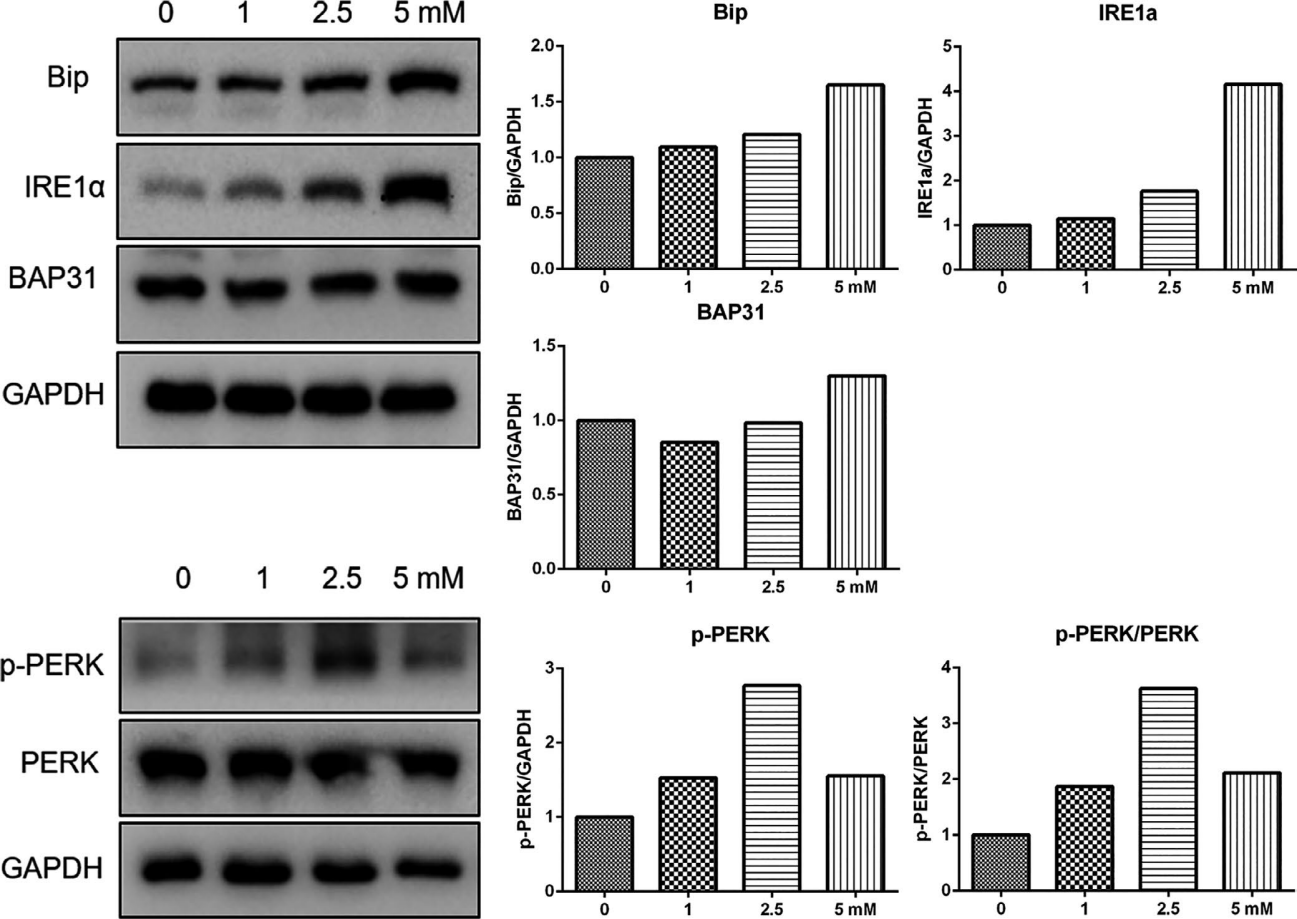
Expression of endoplasmic reticulum (ER) stress‐related genes were increased in cells that underwent melatonin therapy for 24 h. Expression of ER stress‐related genes as evaluated by Western blot analysis

The ER stress has been shown to mediate apoptosis, necroptosis and autophagy in a wide range of biological processes.^12^, ^13^ Therefore, in the present study, the expression of ER stress‐related genes was determined via RT‐qPCR (Figure S7). Our data showed that the expression of CHOP and IRE1a mRNA was significantly enhanced in cells treated with 2.5 and 5.0 mmol/L melatonin. In addition, the expression of spliced XBP1 was significantly induced by treatment with 2.5 and 5.0 mmol/L melatonin, thereby indicating that melatonin triggered ER stress dependent on the melatonin concentration used. To further confirm this gene expression profile, Western blot was also performed (Figure 4), which showed that the expression of Bip and IRE1a protein was significantly increased by melatonin treatment. Especially, the phosphorylation of PERK protein was markedly elevated in cells that received melatonin treatment. In previous studies, it was indicated that BAP31 is involved in the regulation of ER stress, and our findings by Western blot analysis showed that the expression of BAP31 was slightly up‐regulated in cells treated with 5.0 mmol/L melatonin. To further determine the role of the induction of ER stress in the antitumour effect of melatonin, the IRE1a inhibitor STF‐083010 were applied. Results of the CCK8 assay showed that the survival rate of cancer cells was suppressed by STF‐083010 treatment (Figure S4E). In addition, the melatonin induced up‐regulation of LC3‐II was suppressed by STF‐083010 treatment (Figures S8 and S9).

### 3.3 Melatonin targeted the RAS‐RAF‐MAPK signalling pathway

Ras‐Raf‐MAPK signalling is a key pathway in the regulation of cancer development and progression. To further determine the role of melatonin in gastric cancer therapy, in the present study, the RAS/RAF/MAPK signalling pathway was studied. Results of the RT‐qPCR indicated that the expression of H‐Ras was significantly increased in cells that were exposed to 2.5 and 5.0 mmol/L melatonin (Figure S10A). However, the expression of N‐Ras was significantly decreased in cells that were treated with 5.0 mmol/L melatonin (Figure S10A). Western blot analysis revealed that after melatonin treatment, c‐Raf phosphorylation increased in a dose‐dependent manner (Figure S10B). The expression of MEK2 mRNA was significantly enhanced in cells treated with 2.5 and 5.0 mmol/L melatonin (Figure S10A) and Western blot analysis confirmed that MEK1/2 phosphorylation was increased in a dose‐dependent manner (Figure S10B). Furthermore, the phosphorylation of p90Rsk was nearly non‐detectable in control cells and in 1.0 mmol/L melatonin‐treated cells, however, its phosphorylation reached high levels when the melatonin‐treated concentration reached 5.0 mmol/L (Figure S10B). Additionally, melatonin exposure resulted in the phosphorylation of ERK, which gradually increased in a dose‐dependent manner (Figure S10C). Exposure of cancer cells to melatonin also resulted in an increase in the expression of p38 MAPK. Especially, treatment with 5.0 mmol/L melatonin induced the phosphorylation of p38 MAPK protein (Figure S10C). To summarize, these results suggested that the Ras‐Raf‐MAPK signalling pathway was activated after melatonin treatment. However, the extracellular signal‐regulated kinase inhibitor U0126 did not augment the proliferation‐inhibiting effect of melatonin (Figure S4A and S4B). Furthermore, Z‐VAD‐FMK was shown to increase the ratio of p‐ERK/ERK and p‐p38 MAPK/p38 MAPK in the cells treated with 2.5 mmol/L melatonin (Figure S2) but decreased their expression in 5.0 mmol/L melatonin‐treated cells (Figure S3), suggesting that the role of Ras‐Raf‐MAPK signalling pathway in melatonin induced apoptosis depend on the concentration of melatonin.

### 3.4 RNA‐seq transcriptome of mRNA expression in gastric cancer cells after melatonin treatment

**FIGURE 5:**
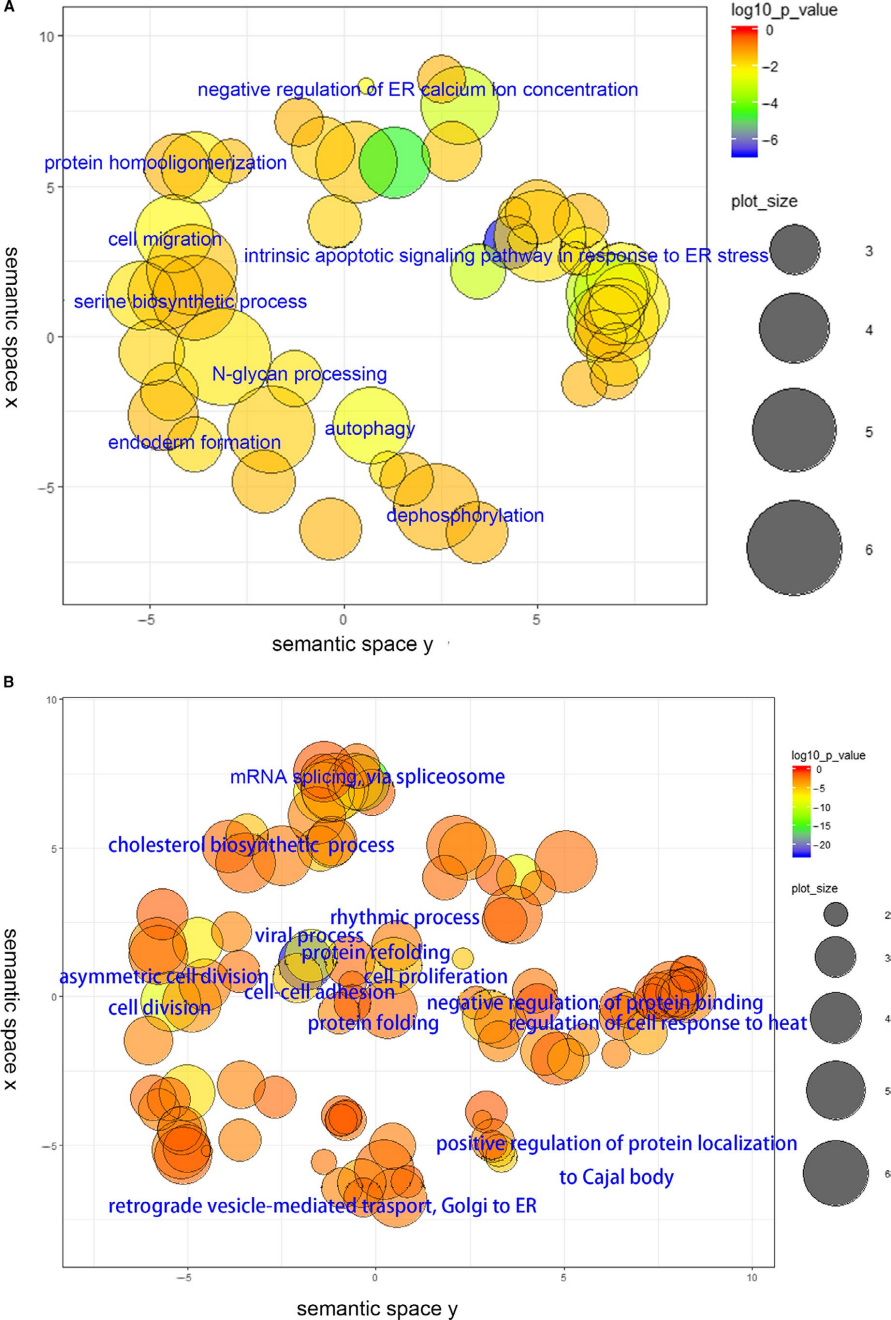
Results of RNA‐seq analysis in melatonin‐treated cells (5 mmol/L for 24 h). (A) Cluster of up‐regulated genes. (B) Cluster of down‐regulated genes

**FIGURE 6:**
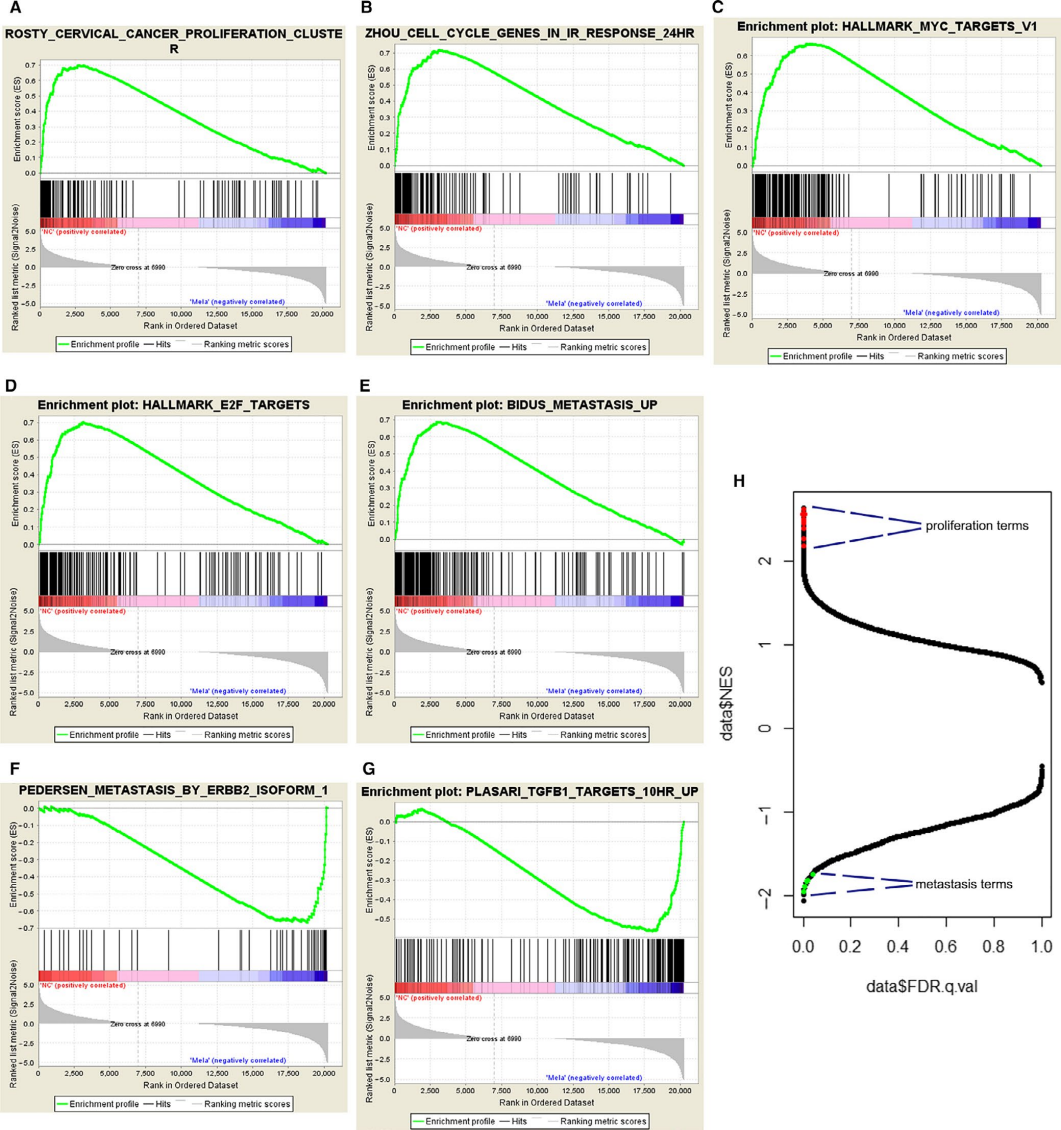
GSEA analysis was performed on RNA‐seq data. (A) Cancer proliferation‐related pathways were down‐regulated in melatonin‐treated cells. (B) The cell cycle in irradiation response‐related pathways were down‐regulated in melatonin‐treated cells. (C) Myc‐associated pathways were down‐regulated in melatonin‐treated cells. (D) E2F‐related pathways were down‐regulated in melatonin‐treated cells. (E) Metastasis (BIDUS)‐associated pathways were down‐regulated in melatonin‐treated cells. (F) ERBB2 isoform‐associated metastasis pathways were enriched in melatonin‐treated cells. (G) TGF‐related pathways were enriched in melatonin‐treated cells. (H) Many proliferation‐related items were decreased and several metastasis‐associated items were enriched in melatonin‐treated cells

To further identify the underlying mechanisms involved in the antitumour effects of melatonin in gastric cancer, RNA‐seq analysis was performed in cells that was treated with 0 and 5 mmol/L melatonin. The gene expression profiles of mRNA are presented in Figure S11A and S11B. A total of 1562 and 1466 genes were up‐ and down‐regulated, respectively, in melatonin‐treated cells compared to control cells. To determine the role of differentially expressed mRNAs, Gene Ontology (GO) enrichment analysis was conducted. As presented in Figure 5A,B, many functional groups were categorized in GO enrichment of significantly up‐ and down‐ expressed mRNAs, and the top functional groups are annotated in these figures. As expected, a large group of genes that is related to ER stress and autophagy was enhanced after melatonin treatment, and in the melatonin group many genes associated with cell proliferation were decreased. Surprisingly, a cluster of genes related to cell migration was increased after melatonin treatment. GSEA further confirmed that pathways associated with proliferation (Figure 6A), cell cycle (Figure 6B), Myc targets (Figure 6C), E2F targets (Figure 6D) and metastasis (Figure 6E) were down‐regulated in the melatonin group. However, a significant enrichment of pathways related to ERBB2 induced metastasis (Figure 6F) and TGFβ1 targets (Figure 6G) was found in melatonin‐treated cells. In general, gene clusters that were significantly down‐regulated in melatonin‐treated cells, were associated with proliferation (Figure 6H). In contrast, several gene clusters that were significantly up‐regulated in melatonin‐treated cells were associated with metastasis. These results might suggest that most cancer cells suffered cell death or transitioned into a static condition, however, the remaining cells would gain the properties of metastasis.

### 3.5 Melatonin combined with thapsigargin treatment enforced gastric cancer cell apoptosis

**FIGURE 7:**
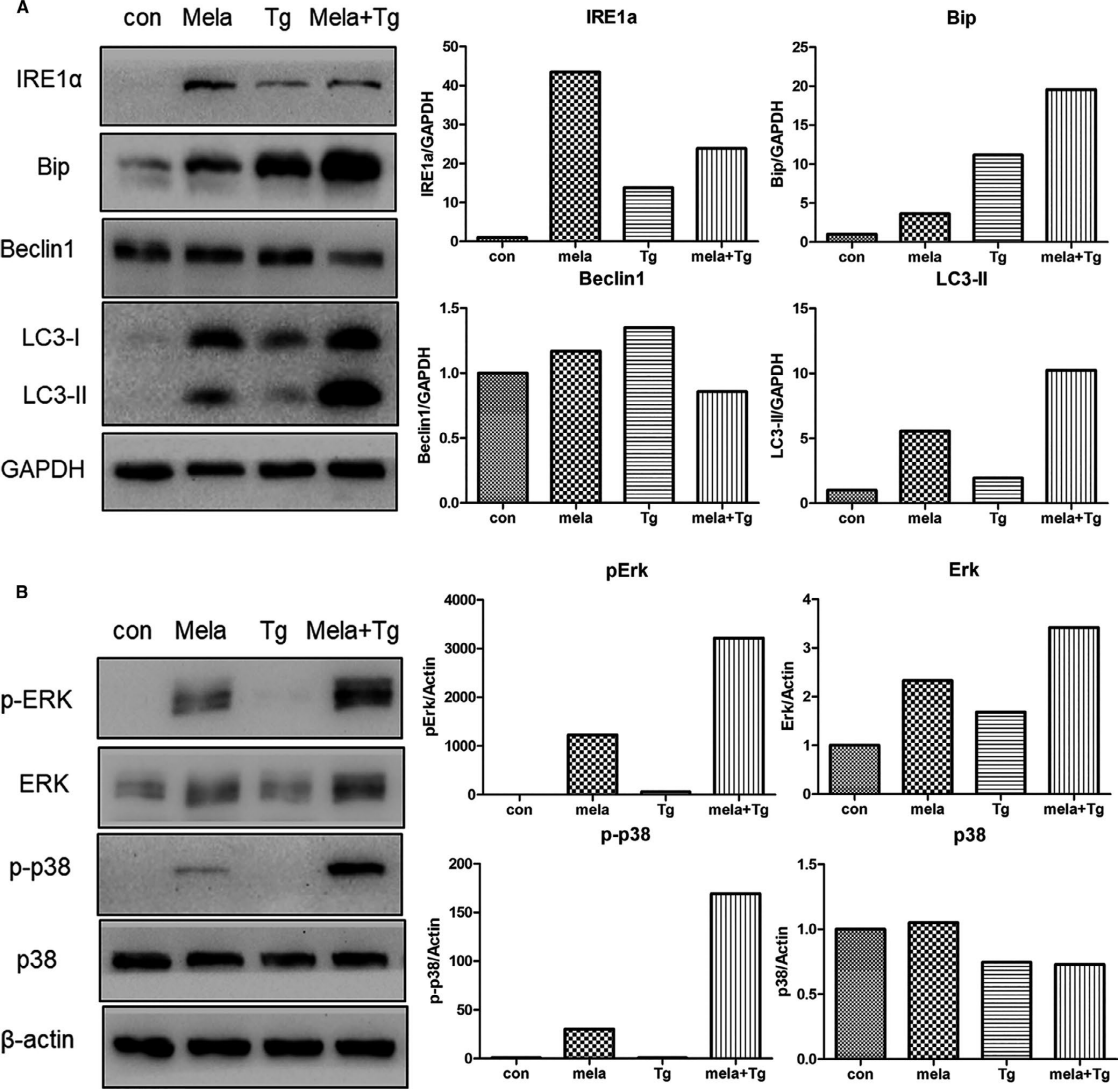
Effects of combinatorial treatment with melatonin (mela, 2.5 mmol/L) and thapsigargin (Tg, 5 μg/mL) for 24 h in gastric cancer cells. (A) Expression of endoplasmic reticulum stress‐ or autophagy‐related genes as determined by Western blot analysis. (B) Expression of MAPK signalling genes as evaluated by Western blot analysis

Considering that the remaining cells after melatonin therapy could potentially metastasize, we aimed to further induce the death of gastric cancer cells via combinational treatment. Previous results revealed that ER stress was significantly induced after melatonin exposure; therefore, we examined the effect of combining melatonin with thapsigargin, an inducer of ER stress. Results of flow cytometry indicated that a combination of melatonin and thapsigargin triggered a higher apoptotic rate when compared to treatment with melatonin alone (Figure S12A). In addition, treatment with a combination of melatonin and thapsigargin enhanced the number of cells in G0/G1 phase of the cell cycle (Figure S12B). Gene expression analysis indicated that the expression of IRE1a in cells that underwent combinatorial treatment was higher compared to individual treatment with thapsigargin. Furthermore, the expression of Bip in cells that underwent combinatorial treatment was higher compared to cells that received individual thapsigargin, individual melatonin or control treatment (Figure 7A). Previous studies revealed the relationship between ER stress and autophagy, and in this study, we demonstrated that melatonin treatment played a role in autophagy. Thus, the expression of autophagy marker genes Beclin1 and LC3 was analysed. As plotted in Figure 7A, when comparing individual treatment with melatonin or thapsigargin, the expression of beclin1 in gastric cancer cells that underwent combinatorial treatment was low. In contrast, the expression of LC3‐II was higher in cells that received combinatorial treatment when compared to cells that received individual melatonin and thapsigargin treatment. Furthermore, treatment with STF‐083010 further exacerbated the decrease in survival rate induced by combinatorial treatment with melatonin and thapsigargin or another ER stress inducer tunicamycin (Figure S4E). The above‐mentioned results showed the up‐regulation of Ras‐Raf‐MAPK signalling in melatonin‐treated cells, therefore, several components of this signalling pathway were investigated. As shown in Figure 7B, combinatorial treatment with melatonin and thapsigargin further induced the increment of phospho‐Erk1/2 and phospho‐p38. However, STF‐083010 also further promoted the up‐regulation of phosphor Erk1/2 in melatonin‐treated cells. Thus, it is possibly indicating that activation of the Ras‐Raf‐MAPK signalling pathway participated in the melatonin‐induced proliferation inhibiting independent of ER stress.

## 4 DISCUSSION

In the present study, we observed that melatonin inhibited the proliferation of gastric cancer in a dose‐dependent manner. In GSEA analysis, many gene items related to proliferation were shown to be down‐regulated by melatonin therapy. Previous studies have well‐documented the inhibitory effect on gastric cancer cell proliferation after melatonin treatment.^14^, ^15^ In addition to proliferation, melatonin also modulated the expression of several types of cell death in a dose‐dependent manner. The results of RNA‐seq showed that many apoptosis‐related genes expression was increased by melatonin treatment. In addition, cleaved caspase 3 and the ratio of Bax/Bcl2 was increased in cells following melatonin treatment. The relationship between melatonin treatment and apoptosis has been documented in previous studies.^16^ However, 5 mmol/L melatonin induced lower cleaved caspase 3, cleaved caspase 9 and ratio of Bax/Bcl2 than 2.5 mmol/L melatonin, suggesting that many other cell death mechanisms were triggered by high concentration of melatonin. Thus, we investigated the expression of necrosis‐associated genes after melatonin therapy. In line with the results of previous studies, we showed that increased expression of MLKL, RIPK1 and PARP in cells after melatonin treatment,^17^ indicating that necroptosis was possibly induced by melatonin treatment. In addition, the increased expression of cleaved PARP in cells exposed to melatonin suggested that another type of controlled necrosis, named parthanatos might also participate in melatonin therapy. Parthanatos is a process that is dependent on the activity of PARP.^18^ The cleavage of PARP is catalysed by caspase 3,^19^ the cleavage type which was also enhanced by melatonin treatment in the present study. Autophagy was significantly induced by melatonin treatment by enhancing the expression of ATG3, ATG5 and Beclin1. LC3, a key protein that contributes to major steps of autophagy, is considered an autophagy marker. Melatonin induced the turnover of LC3‐I into LC3‐II in a dose‐dependent manner. In addition, PI3K‐Akt‐mTOR signalling participates in regulating autophagy and cell viability.^20^ Activation of the PI3K‐Akt‐mTOR pathway is closely associated with the inhibition of autophagy.^21^ In the present study, phosphorylation of Akt and mTOR was decreased in cells that received melatonin treatment, further confirming that autophagy was triggered by melatonin treatment. Furthermore, in the present study, we demonstrated that the increase in Bax/Bcl2 ratio in melatonin‐treated cells was reduced by 3‐MA or CQ therapy, possibly indicating that inhibition of autophagy weakened the pro‐apoptosis effect of melatonin. The cleaved form of PARP, which in melatonin treated cells was further enhanced by 3‐MA or CQ therapy, suggested that the inhibition of autophagy would lead to further increased parthanatos by melatonin. Autophagy might possibly act as a switch for different types of cell death after melatonin exposure. In fact, cells received 5 mmol/L melatonin treatment showed a relative lower expression level of Bax, Bcl2, cleaved caspase 3 and cleaved caspase 9 but higher expression level of LC3‐II and cleaved PARP than those in cells with 2.5 mmol/L melatonin treatment, possibly suggesting that many more cells suffered necroptosis and/or autophagic cell death when treated concentration increasing from 2.5 to 5.0 mmol/L. Furthermore, in the present study, we found that Ras‐Raf‐MAPK signalling was activated by melatonin treatment. The Ras‐Raf‐MAPK signalling pathway plays an important role in antiproliferative events, including apoptosis, autophagy and senescence.^22^, ^23^, ^24^ The Ras‐Raf‐MAPK pathway induced mitochondrial cytochrome c release or activate caspase 8, processed cell cycle arrest or triggered autophagic vacuolization, thereby promoting either intrinsic or extrinsic apoptotic pathways. The intrinsic pathway of apoptosis is composed of releasing proapoptotic factors, such as cytochrome c, from the mitochondria in the cytoplasm, and activates caspase 9, which in turn activates caspase 3 or 7.^22^ In this study, cleaved caspase 3 and 9 were elevated after melatonin treatment, and our findings indicated that melatonin treatment possibly triggered the intrinsic apoptotic pathway.

The ER modulates the adaptive capacity of tumour cells to varied cell‐intrinsic and cell‐extrinsic stresses by coordinating many fundamental cellular processes. Normal ER functions can be disrupted by various physiological or pathological stimuli, thereby inducing the accumulation of unfolded or misfolded proteins in the ER lumen, leading to a condition named ‘ER stress’.^16^, ^25^ ER stress resulted in an altered expression level and activities of key regulators of cell survival. Cell death programmes would activate to eliminate damaged cells when ER stress fails to restore homeostasis. ER stress and its downstream signalling, including autophagy and apoptosis, play an important role in chemotherapy. In this study, the spliced form of XBP1 was enhanced in cells that underwent melatonin treatment, thus indicating that ER stress was triggered by melatonin treatment. In previous studies, it was indicated that XBP1 mRNA splicing could lead to the induction of autophagy via down‐regulation of Beclin1.^26^ When applying the ER stress inducer thapsigargin, we found that the increased expression of Beclin1 and LC3‐II accompanied the induction of ER stress. STF‐083010, a novel small‐molecule inhibitor of IRE1a, has shown significant antitumour activity in several type of cancers.^27^, ^28^, ^29^ In the present study, STF‐083010 was shown to further inhibit the proliferation of cells subjected to combinatorial treatment with melatonin and thapsigargin or tunicamycin. These finding possibly indicated that ER stress induced by melatonin treatment was a protective strategy for cancer cells to escape from death.

Many cancer‐associated deaths are related to the metastasis of cancer cells. Tumour cells have the ability of migrating out of the primary tumour to invade into the surrounding tissue and establish metastases at distant organs.^25^, ^26^ When considering the impact of melatonin therapy on cell death, it is suggested that melatonin therapy could inhibit metastasis. However, GSEA analysis suggested that the expression of several pathways related to metastasis, including TGFβ1 targets, were activated by treatment with 5 mmol/L melatonin. Thus, cancer cells that survive high concentrations of melatonin exposure may possibly gain the capability of metastasis. In previous reports, it has been documented that several types of chemotherapy suppressed the proliferation of primary tumours and enhanced drug resistance and metastasis. For example, paclitaxel, a widely used anticancer drug, has been found to promote breast cancer metastasis in a Toll‐like receptor 4 (TLR4) dependent manner.^30^ To conquer such risks of drug resistance and metastasis, combinatorial treatment would be a useful therapeutic strategy. Combinatorial therapy has the advantage of targeting multiple pathways and reducing side effects, and presents more promising therapeutic outcomes. In this study, we confirmed that ER stress could be promoted by melatonin treatment. In addition, caspase‐mediated cell death could be triggered by prolonged and severe pharmacological ER stress.^31^ To improve the therapeutic effect of melatonin, cancer cells were co‐treated with melatonin and the ER stress inducer thapsigargin. As expected, both the apoptotic rate and G0/G1 cell cycle arrest were increased in cells that received combinatorial treatment of melatonin and thapsigargin via triggering ER stress. In fact, melatonin has been shown to exhibit more promising antitumour effect when combining with a wide range of drugs, including dabrafenib,^32^ shikonin,^33^ valproic acid ^17^ and cisplatin.^34^ These evidences suggested that combinatorial treatment is an effective approach to enhance the capability of melatonin to combat cancer.

The Ras‐Raf‐MAPK pathway has been shown to participate in melatonin‐induced apoptosis in several cancers.^17^, ^35^ However, the survival rate of melatonin‐treated cells was not altered by U0126 (a selective inhibitor of MEK1 and MEK2) treatment. Z‐VAD‐FMK in combination with 2.5 mmol/L melatonin increased the phospho‐Erk1/2 and phospho‐p38, but Z‐VAD‐FMK in combination with 5 mmol/L melatonin decreased the phospho‐Erk1/2 and phospho‐p38. Furthermore, both thapsigargin and STF‐083010 could both promote the increment of the phospho‐Erk1/2 in the cells treated with melatonin. These evidences possibly suggested that activation of Ras‐Raf‐MAPK pathway is a downstream event of melatonin‐induced proliferation inhibition. Previous reports have shown that ERKs acted as a double‐edged sword in cancers which can exert antitumour function by regulating apoptosis, differentiation and senescence, or trigger pro‐survival pathways contributing to cell proliferation and migration.^36^ Further studies should be posted to investigate the feedback circuitries of Ras‐Raf‐MAPK pathway to broaden the application of melatonin in cancer treatment.

The present study further revealed the molecular fluctuation in the cells with melatonin treatment. ER stress was a possible linkage under melatonin‐induced apoptosis and autophagy. Furthermore, high concentration of melatonin might increase the risk of metastasis. Utilizing the synergistic effect of combinatorial treatment would be help to enhance therapeutic applications and avoid potential side effects of melatonin therapy.

## CONFLICT OF INTEREST

The authors declare that they have no competing interests.

## AUTHOR CONTRIBUTIONS

**Yongye Huang:** Conceptualization (equal); Funding acquisition (equal); Project administration (equal); Writing‐original draft (equal); Writing‐review & editing (equal). **Kexun Yuan:** Investigation (equal). **Meifang Tang:** Investigation (equal). **Jiaming Yue:** Investigation (equal). **Lijun Bao:** Investigation (equal). **Shuang Wu:** Investigation (equal). **Yanxin Zhang:** Writing‐review & editing (equal). **Yin Li:** Investigation (equal). **Yihang Wang:** Investigation (equal). **Xu Ou:** Investigation (equal). **Jiaxin Gou:** Investigation (equal). **Qi Zhao:** Data curation (equal); Formal analysis (equal); Writing‐review & editing (equal). **Lin Yuan:** Conceptualization (equal); Writing‐original draft (equal); Writing‐review & editing (equal).

## Supporting information

Supplementary Material

## Data Availability

The raw data of RNA‐seq has been uploaded into SRA (PRJNA680660).
