# Supplementary material for: Melatonin inhibiting the survival of human gastric cancer cells under ER stress involving autophagy and Ras‐Raf‐MAPK signalling
Source: J Cell Mol Med. 2020 Dec 25;25(3):1480–92. doi: 10.1111/jcmm.16237 (PMC7875909; doi:10.1111/jcmm.16237)

Supplemental Materials

melatonin inhibiting the survival of human gastric cancer cells under ER stress involving autophagy and Ras-Raf-MAPK signaling

Running Title: melatonin treatment for gastric cancer

Yongye Huang [1], Kexun Yuan [1], Meifang Tang [1], Jiaming Yue [1], Lijun Bao[1], Shuang Wu [1], Yanxin Zhang[2], Yin Li[1], Yihang Wang[1], Xu Ou[1], Jiaxin Gou[1], Qi Zhao [3,] *, Lin Yuan [4,] *

[1] College of Life and Health Sciences, Northeastern University, Shenyang, 110169, China;

[2] National Academy of Innovation Strategy, Beijing, 100038, China;

[3] School of Computer Science and Software Engineering, University of Science and Technology Liaoning, Anshan, 114051, China;

[4] Institute of Health Science, China Medical University, Shenyang 110122, China.

[*] To whom correspondence should be addressed: Tel: +86-24-83656116. Fax: +86-24-83656116. E-mail: zhaoqi@lnu.edu.cn (Qi Zhao), penguinyl@163.com (Lin Yuan).

1.Spplemental procedure

1.1 Proliferation assay

Cell proliferation was determined using Cell Counting Kit-8 (CCK-8; Bimake, Houston, TX, USA), which is based on a water-soluble tetrazolium salt WST-8 that produces a water-soluble formazan dye upon reduction in the presence of an electron mediator. The amount of the formazan dye produced by dehydrogenases in the cell is directly proportional to the number of live cells.

In briefly, cells were seeded in 96-well plates at a density of 4,000 cells/well and exposed to various drugs for different time points. Subsequently, 10 μL of CCK-8 reagent was added into each well and cells were incubated for an additional 2h. The optical density (OD) was measured at a wavelength of 450 nm using a microplate reader.

1.2 Annexin V-FITC/PI assay

Cellular apoptosis was determined using Annexin-V/PI double staining. Annexin V-FITC was used to stain the membrane of the early-stage apoptotic cells (green), and propidium iodine (PI) was used to stain the nuclei of late apoptotic or necrotic cells (red). After treatment with melatonin, cells were washed with PBS, incubated at room temperature in 100 μL binding buffer containing Annexin V-FITC and PI for 15 min in the dark. Next, 400 μL of binding buffer was added to a final volume of 500 μL. Cells (10,000 singlets) were then analyzed via Fortessa flow cytometer (BD Biosciences).

1.3 Deep sequencing analysis

For deep sequencing analysis, total RNA was extracted from gastric cancer cells that were treated with or without melatonin. The quality of RNA was assessed by a Qubit 3.0 Fluorometer, an Agilent 2100 Bioanalyzer and a NanoDrop spectrophotometer. Library preparation and Illumina sequencing analysis were completed by Genecreate (Wuhan, China).

2. Supplemental figures

Figure S1. Expression of apoptosis-related genes as evaluated by RT-qPCR. Data were presented as the mean ± SEM of duplicate experiments. *p<0.05, **p<0.01 and ***p<0.001, indicated significant differences when compared with the control group.

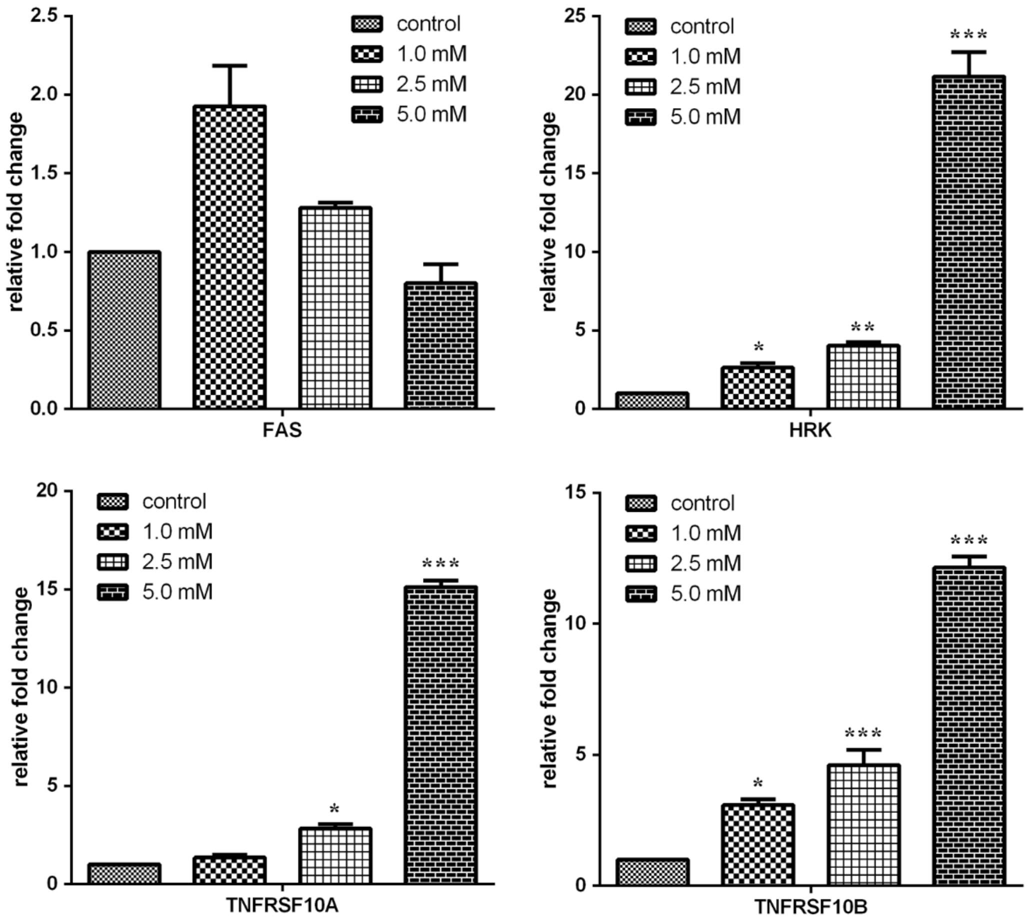

Figure S2. Gene expression as determined by Western blot analysis in cells treated with melatonin (mela2.5, 2.5 mM) and Z-VAD-FMK (ZVAD, 10 μM).

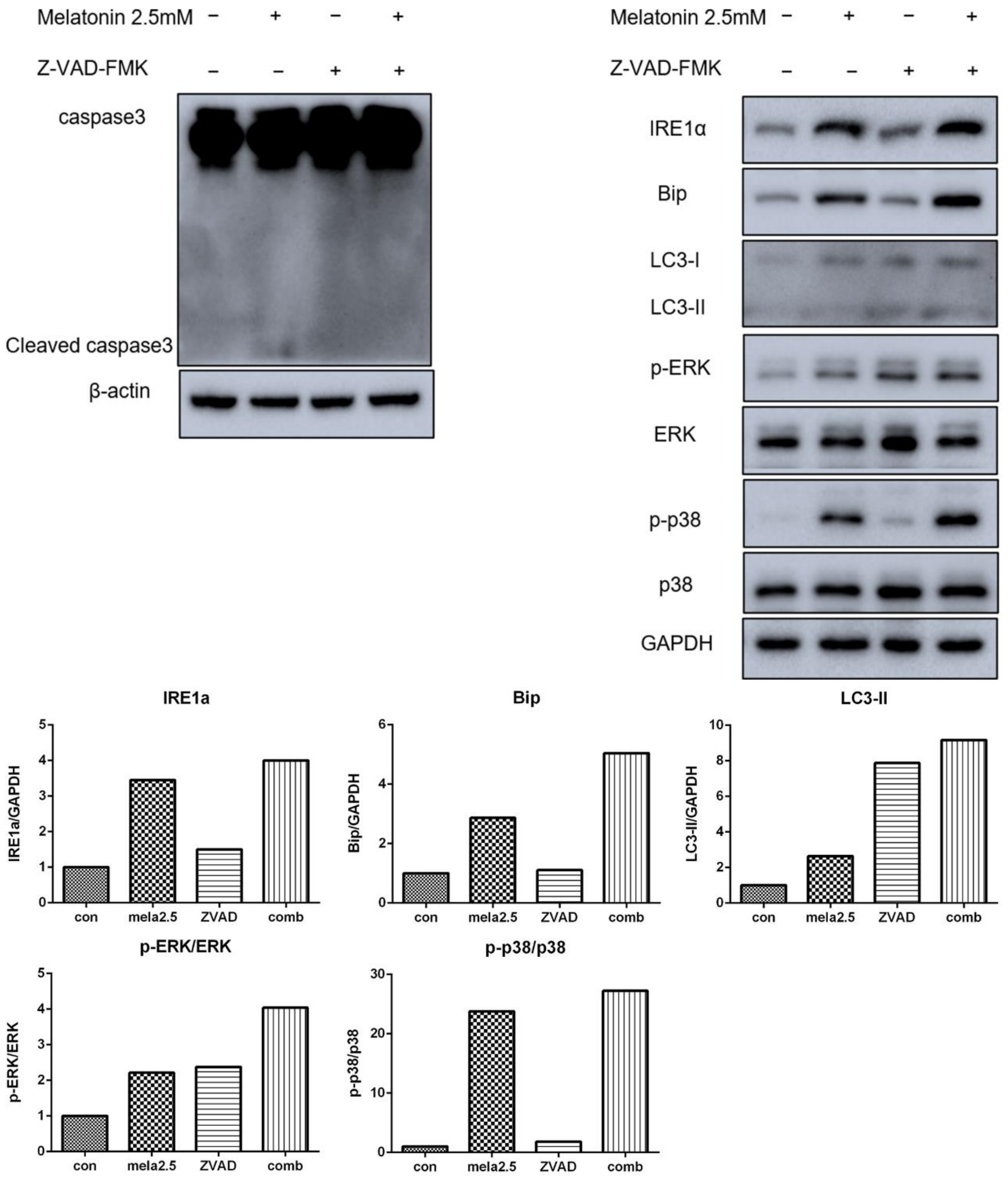

Figure S3. Gene expression as determined by Western blot analysis in cells treated with melatonin (mela5, 5 mM) and Z-VAD-FMK (ZVAD, 10 μM).

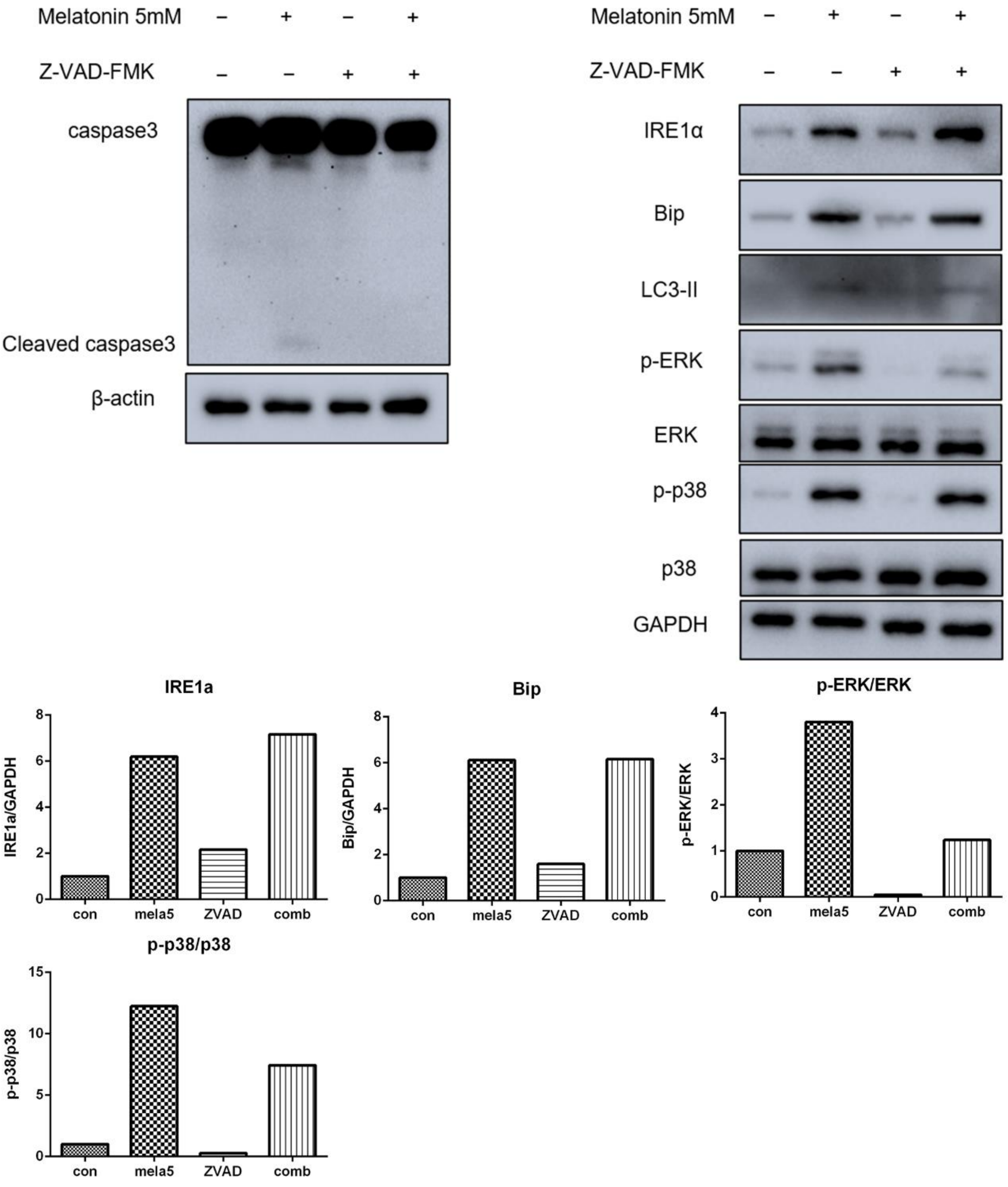

Figure S4. The survival rate of gastric cancer cells as determined by CCK8 assay. (A) Cells were pre-treated with U0126 (10 μM) or Z-VAD-FMK (20 μM) for 2 h, followed by melatonin treatment (Mela, 2.5 mM) for 24 h. (B) Cells were pre-treated with U0126 (10 μM) or Z-VAD-FMK (20 μM) for 2 h, followed by melatonin treatment (Mela, 5 mM) for 24 h. (C) Cells were co-treated with melatonin (Mela,

2.5 mM) and U0126 (10 μM)/ Z-VAD-FMK (20 μM)/ 3-MA (50 mM)/ CQ (20 μM) for 24 h. (D) Cells were co-treated with melatonin (Mela, 5 mM) and U0126 (10 μM)/ Z-VAD-FMK (20 μM)/ 3-MA (50 mM)/ CQ (20 μM) for 24 h. (E) Cells were co-treated with melatonin (Mela, 5 mM) and STF-083010 (STF, 50 μM)/ thapsigargin (Tg, 5 μg/mL)/ Tunicamycin (Tu, 1 μg/mL) for 24 h.

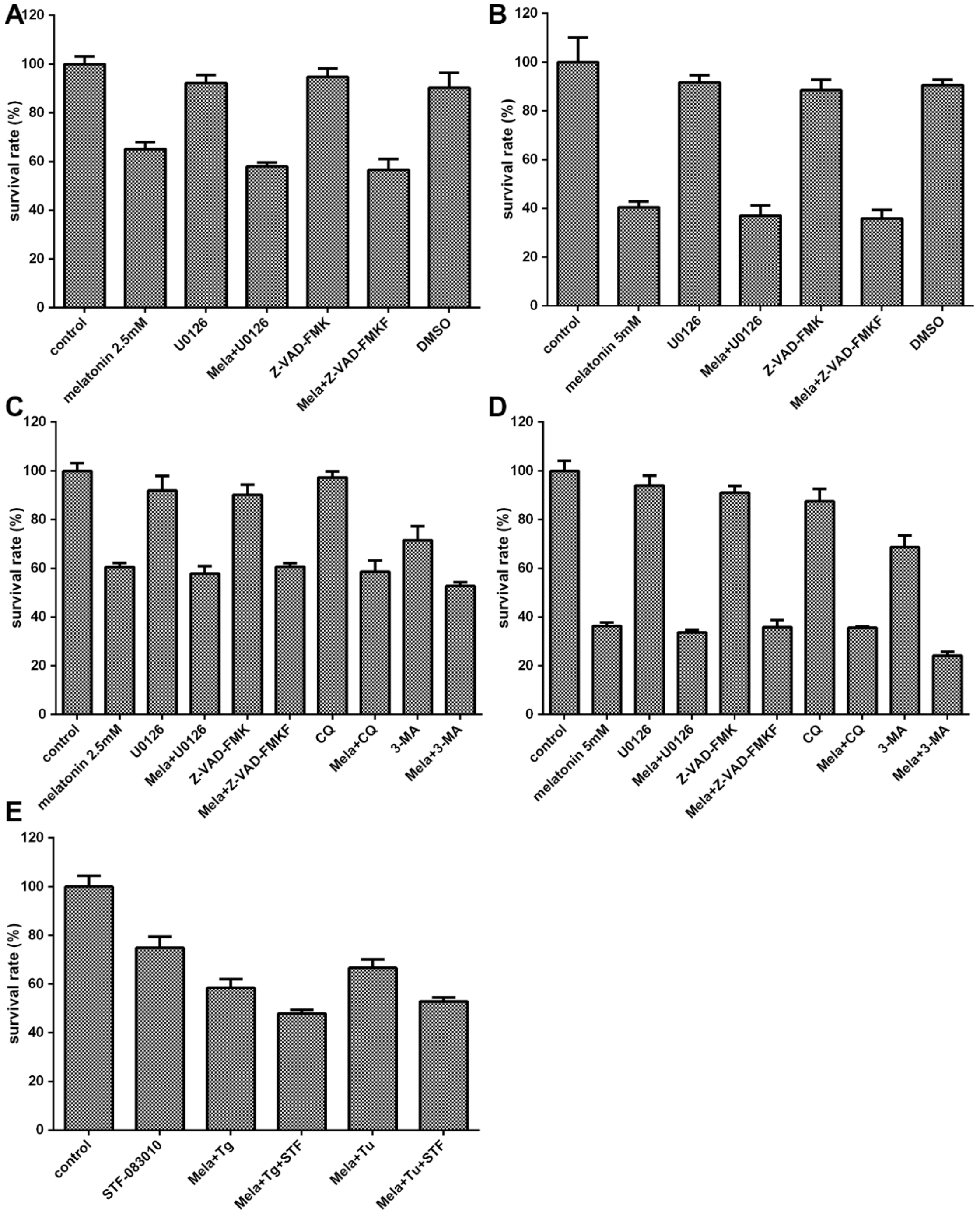

Figure S5. Effect of melatonin on the expression of necrosis-associated genes. (A) RT-qPCR analysis of MLKL, RIPK1, and PARP1 in cells after 24 h of melatonin treatment. (B) Western blot analysis of PARP in cells after 24 h of melatonin treatment. Values represent the mean ± SEM. ** *p*<0.01 versus control, and ****p* <0.001 versus control.

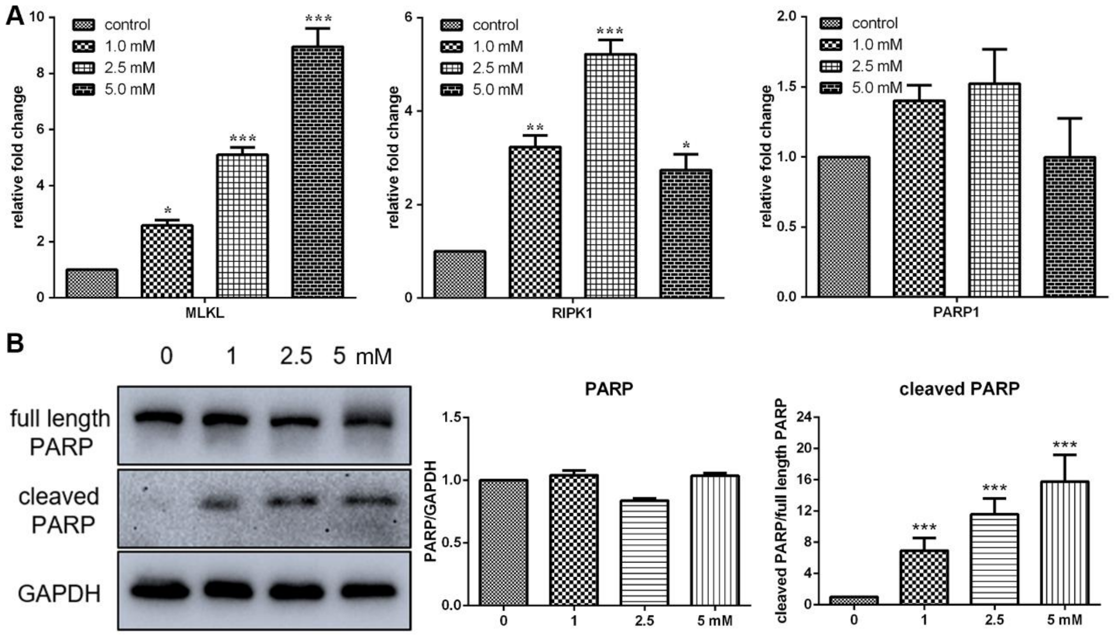

Figure S6. Treatment with melatonin (24 h) induced autophagy in cancer cells by inhibiting Akt/mTOR phosphorylation. (A) Expression of autophagy-related genes as evaluated by RT-qPCR. (B) Expression of LC3 as evaluated by Western blot analysis. (C) Expression of LC3 Akt/mTOR signal as evaluated by Western blot analysis. Data are presented as the mean ± SEM of duplicate experiments. *p<0.05 and **p <0.01, indicate significant differences when compared with the control group.

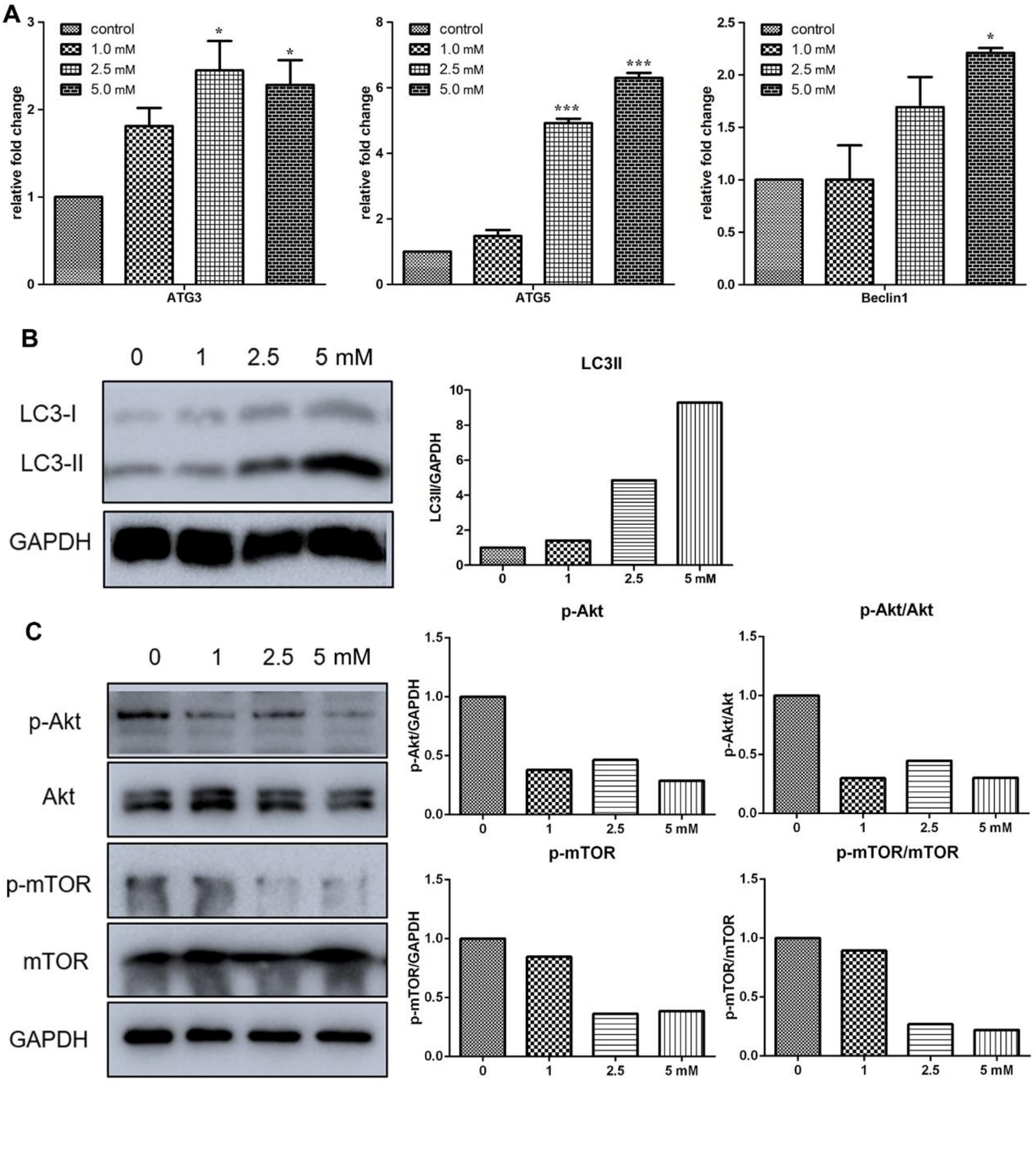

Figure S7. Expression of ER stress-related genes as determined by RT-qPCR. Values represent the mean ± SEM. *p <0.05 versus control, ***p* <0.01 versus control and ****p* <0.001 versus control.

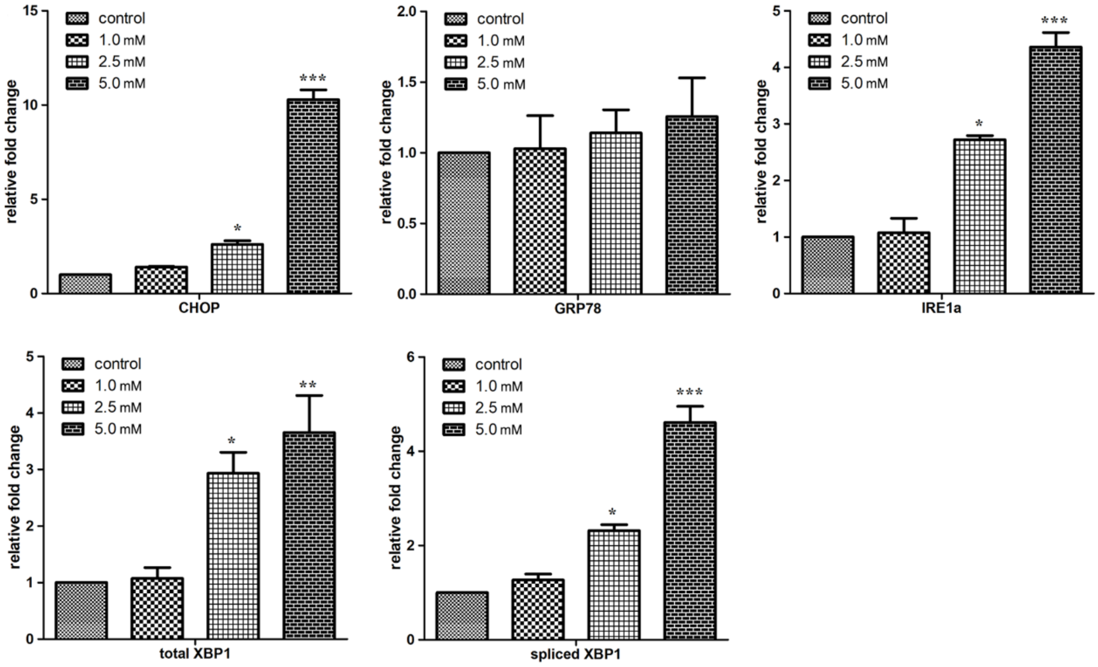

Figure S8. Gene expression as determined by Western blot analysis in cells treated with melatonin (mela2.5, 2.5 mM) and STF-083010 (STF, 50 μM).

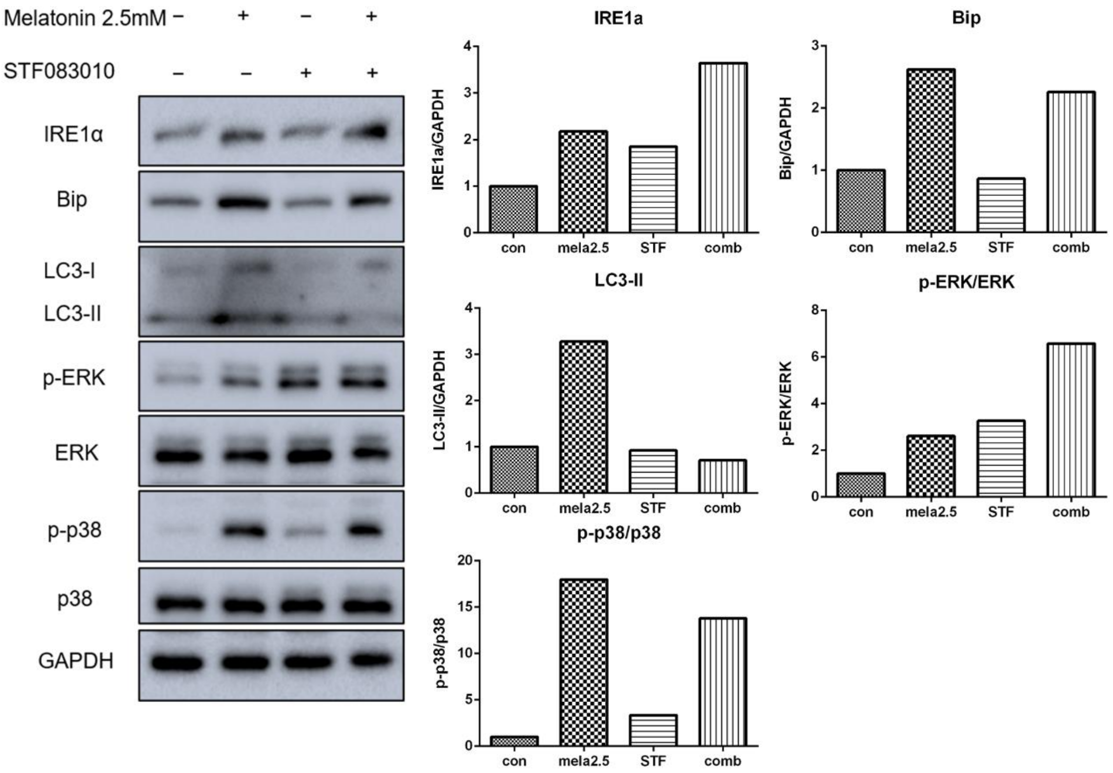

Figure S9. Gene expression as determined by Western blot analysis in cells treated with melatonin (mela5, 5 mM) and STF-083010 (STF, 50 μM).

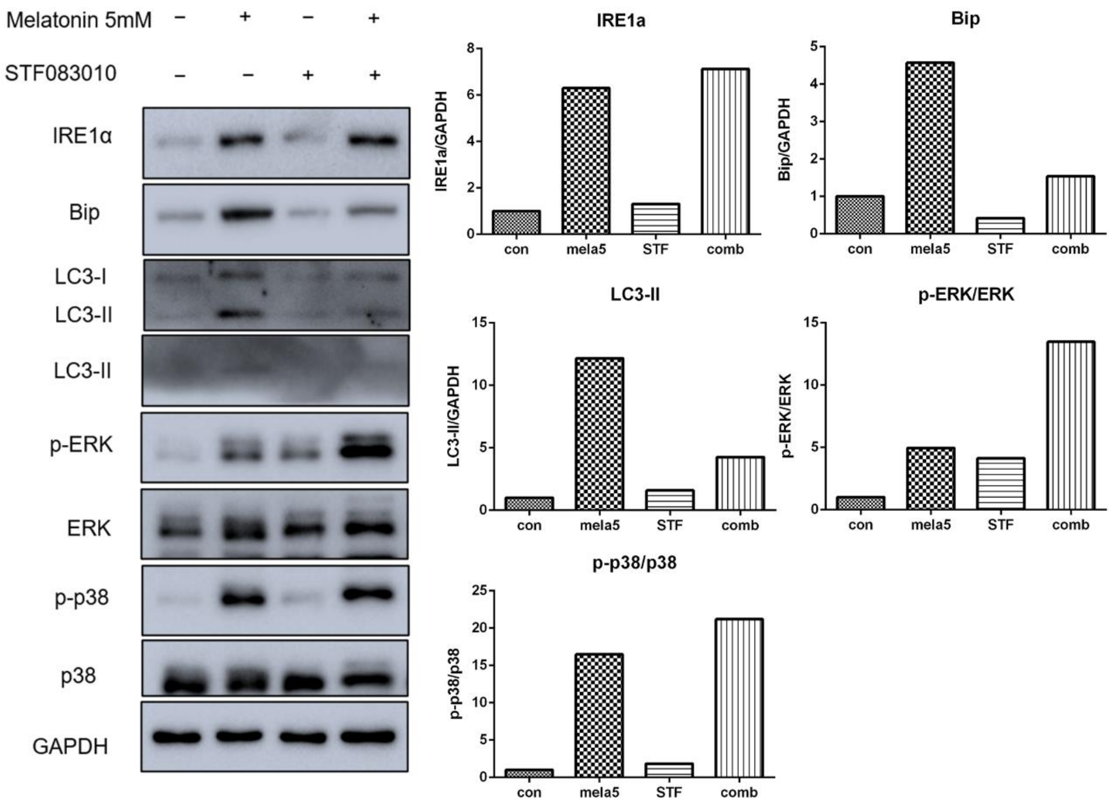

Figure S10. Up-regulation of the Ras/Raf/MEK/ERK signaling pathway in cells after 24 h of melatonin-treatment. (A) Expression of H-Ras, N-Ras and MEK2 as determined by RT-qPCR. (B) Phosphorylation of c-Raf, MEK1/2 and p90Rsk as determined by Western blot analysis. (C) Expression of MAPK as evaluated by Western blot analysis. Values represent the mean ± SEM of duplicate experiments. **p*<0.05, ***p* <0.01 and ****p* <0.001, indicate significant differences when compared with the control group.

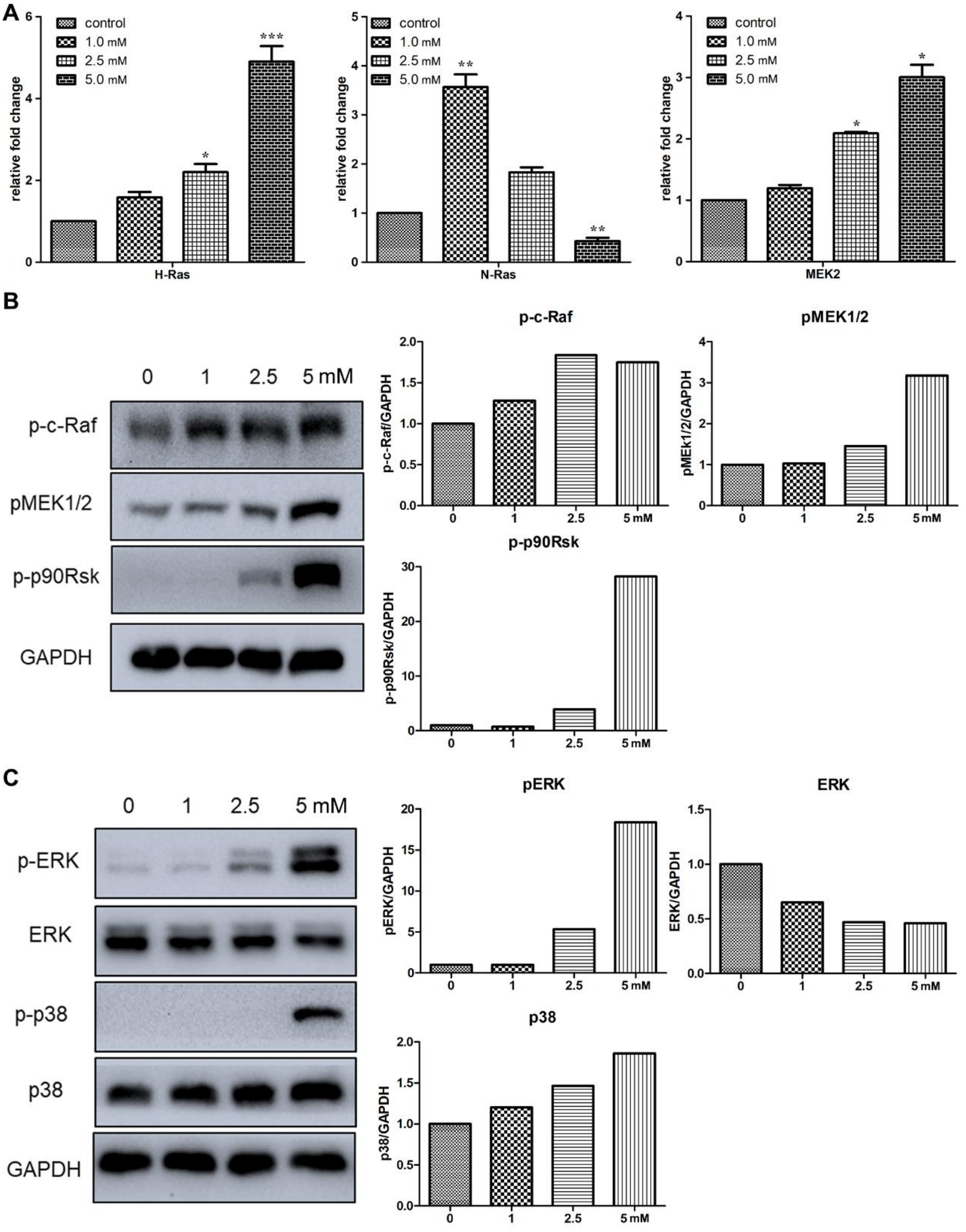

Figure S11. Results of RNA-seq analysis in melatonin treated cells (5 mM for 24 h). (A) Heatmap of differentiated gene expression profile from RNA-seq data. (B) Heatmap of gene expression profile from RNA-seq data.

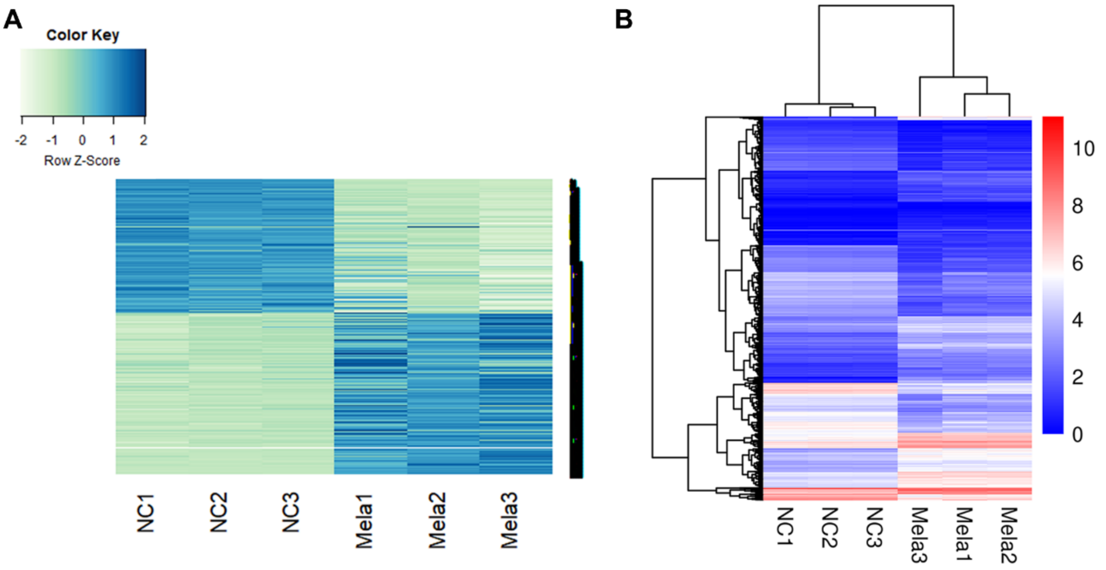

Figure S12. Apoptosis and cell cycle distribution in gastric cancer cells subjected to combinatorial treatment with melatonin (2.5 mM) and thapsigargin (5 μg/mL) for 24 h. (A) Apoptosis was evaluated by flow cytometry analysis using annexin V/PI staining. (B) Cell cycle distribution was determined by flow cytometry analysis using PI staining.

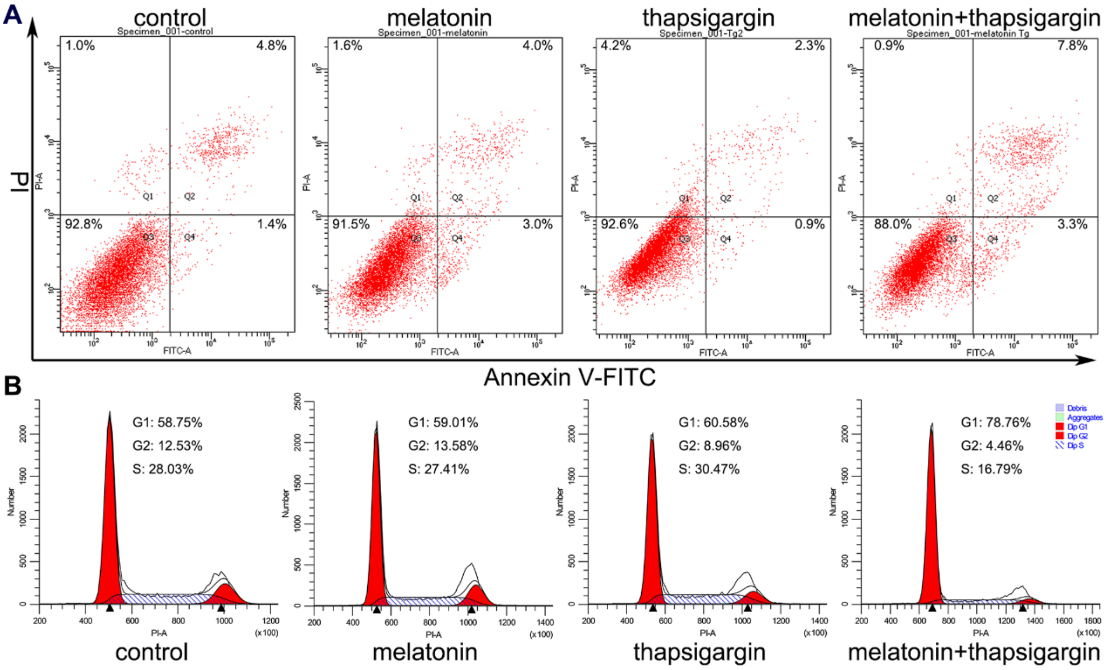

Supplement: Supplementary file 1 — Supplementary Material [file JCMM-25-1480-s001.pdf]
